# Isolation, Expression, and Promoter Analysis of *GbWRKY2*: A Novel Transcription Factor Gene from *Ginkgo biloba*

**DOI:** 10.1155/2015/607185

**Published:** 2015-08-16

**Authors:** Yong-Ling Liao, Yong-Bao Shen, Jie Chang, Wei-Wei Zhang, Shui-Yuan Cheng, Feng Xu

**Affiliations:** ^1^College of Forestry, Nanjing Forestry University, Nanjing, Jiangsu 210037, China; ^2^College of Horticulture and Gardening, Yangtze University, Jingzhou, Hubei 434025, China; ^3^School of Biology and Pharmaceutical Engineering, Wuhan Polytechnic University, Wuhan, Hubei 430023, China

## Abstract

WRKY transcription factor is involved in multiple life activities including plant growth and development as well as biotic and abiotic responses. We identified 28 *WRKY* genes from transcriptome data of *Ginkgo biloba* according to conserved WRKY domains and zinc finger structure and selected three WRKY genes, which are *GbWRKY2, GbWRKY16*, and *GbWRKY21*, for expression pattern analysis. *GbWRKY2* was preferentially expressed in flowers and strongly induced by methyl jasmonate. Here, we cloned the full-length cDNA and genomic DNA of *GbWRKY2*. The full-length cDNA of *GbWRKY2* was 1,713 bp containing a 1,014 bp open reading frame encoding a polypeptide of 337 amino acids. The *GbWRKY2* genomic DNA had one intron and two exons. The deduced *GbWRKY2* contained one WRKY domain and one zinc finger motif. *GbWRKY2* was classified into Group II WRKYs. Southern blot analysis revealed that *GbWRKY2* was a single copy gene in *G. biloba*. Many *cis*-acting elements related to hormone and stress responses were identified in the 1,363 bp-length 5′-flanking sequence of *GbWRKY2*, including W-box, ABRE-motif, MYBCOREs, and PYRIMIDINE-boxes, revealing the molecular mechanism of upregulated expression of *GbWRKY2* by hormone and stress treatments. Further functional characterizations in transiently transformed tobacco leaves allowed us to identify the region that can be considered as the minimal promoter.

## 1. Introduction

*Ginkgo biloba* is the oldest relic plant among extant seed-bearing plants, usually referred to as a “living fossil” [1]. Experiencing million years of complicated climate,* G. biloba* not only shows strong adaptability but also exhibits minor changes in morphology. This phenomenon is possible because* G. biloba* can adapt to changing environments and tolerate harsh conditions [2]. Resistance genes encoding the enzymes in reactive oxygen-scavenging systems, including chloroplast copper/zinc-superoxide dismutase (*GbCuZnSOD*) [3], iron superoxide dismutase (*GbFeSOD*) [4], and manganese superoxide dismutase (*GbMnSOD*) [5], catalase (*GbCAT1*) [6], ascorbate peroxidases (*GbAPX*) [7], peroxidase (*GbPOD1*) [8], as well as dehydrin (*GbDHN*) [2], and* GbASR* [9] response to abscisic acid (ABA), stress, and ripening have been cloned from* G. biloba*. Studies on these genes help us to elucidate the molecular mechanism by which* G. biloba* tolerates severe conditions. Furthermore, stress conditions, such as drought, severe cold, harm, high temperature, and heavy metals, can stimulate accumulation and release of secondary metabolites of flavonoids and terpene lactones from* G. biloba*. Flavonoids and terpene lactones play an important role in improving self-protection, promoting competitive capacity, and coordinating interaction with the environment.

The WRKY family is among the ten largest families of transcription factor (TF) in higher plants and is found throughout the green lineage [10]. The defining feature of WRKY TFs is their DNA binding domain. This is called the WRKY domain containing the conserved WRKYGQK sequence at the N terminal, and as well as containing the WRKY signature it also has a typical zinc finger motif at the C terminal. The zinc finger structure is either C_2_H_2_ (CX_4-5_CX_22-23_HX_1_H) or C_2_HC (CX_7_CX_23_HX_1_C). Based on the number of the WRKY domains and the characteristic of a zinc finger motif, WRKY TFs can be divided into three groups: the first group contains two WRKY domains; the second group and the third group contain one WRKY domain each. The zinc finger motif of the second class is C_2_H_2_, which is the same as that of the first group; the zinc finger motif of the third group is C_2_HC [11].

The WRKY domain binds to what is called W-box (TTGACC/T) in the promoters of target genes. This sequence is the minimal core element necessary for binding of a WRKY protein to DNA [12]. W-box is present in the promoter of many genes related to disease resistance, damage, growth, aging, and WRKY gene, which shows that the WRKY TF is closely related to biotic and abiotic stress responses [13]. Studies have successfully used the WRKY TF to improve the stress resistance of plants. For example, single or cosilence of* NaWRKY3* and* NaWRKY6* can promote the susceptibility of tobacco to herbivore damage by weakening the accumulation of volatile sesquiterpene and jasmonate [14]. The resistance of* Arabidopsis thaliana* to the fungal disease* Botrytis* is also improved by overexpressing* AtWRKY3* and* AtWRKY4* [15]. The tolerance of rice to high temperature and drought can be improved by overexpressing* OsWRKY11*  [16]. The expression level of* MusaWRKY71* is improved after plants suffer from cold, drought, salt, ABA, H_2_O_2_, ethephon (ETH), salicylic acid (SA), and methyl jasmonate (MeJA) in* Musa* spp. [17]. The expression of* HbWRKY1* in latex is induced by ETH and MeJA, while the expression of* HbWRKY1* in the leaf is induced by drought and ABA from* Hevea brasiliensis* Müll. Arg. [18].

The WRKY TF can also participate in the control of the secondary metabolism of plants except in plant defense against exogenous stress. For example, Kato et al. [19] isolated and identified the* CjWRKY1* TF that controls the biosynthesis of berberine from* Coptis japonica*. Ectopic expression of* CjWRKY1* cDNA in* C*.* japonica* protoplasts clearly increased the level of transcripts of all berberine biosynthetic genes examined compared with control treatment, indicating that* CjWRKY1* is a necessary positive regulator to control overall gene expression in berberine biosynthesis. Xu et al. [20] found that* GaWRKY1* gene, which encodes a protein containing a single WRKY domain, could combine with W-box in the promoter region of the key gene encoding (+)-*δ*-cadinene synthase to metabolize terpene and promote sesquiterpene biosynthesis. Ma et al. [21] also isolated* AaWRKY1* gene from glandular secretory trichomes of* Artemisia annua* in which artemisinin is synthesized and sequestered. Transient expression of* AaWRKY1* cDNA in* A. annua* leaves clearly activated the expression of the majority of artemisinin biosynthetic genes, suggesting the involvement of the* AaWRKY1* TF in the regulation of artemisinin biosynthesis. Recently, Li et al. [22] and Sun et al. [23] demonstrated that a* TcWRKY1* gene and a MeJA inducible* PqWRKY1* gene participated in regulation of the biosynthesis of taxol in* Taxus chinensis* and triterpene ginsenoside in* Panax quinquefolius*, respectively.

To date, the WRKY genes have been cloned from many species. A total of 5,936 WRKY genes are recorded in the database of plant TFs. These genes have also been reported in 83 species, such as algae, planus, needle-leaved plant, monocotyledons, and dicotyledons. However, no cloning and the WRKY gene in* G. biloba* has not been found. In this report, a total of 28 unigenes of WRKY transcription factor were identified using next-generation sequencing. Among these unigenes, a MeJA-inducible* WRKY* gene, named* GbWRKY2*, was cloned from* G*.* biloba* using the rapid amplification of cDNA ends (RACE) method.* GbWRKY2* is preferentially expressed in flowers and induced by salinity stress and phytohormones such as SA, ETH, MeJA, and ABA but repressed by heat. Promoter analysis provided the molecular mechanism of upregulation of* GbWRKY2* by phytohormones and abiotic stresses and allowed us to identify the region that can be considered as the minimal promoter. This work represents, to our knowledge, the first functional characterization of a* G*.* biloba* WRKY transcript factor.

## 2. Experimental Section

### 2.1. Plant Materials and Treatments

The 14-year-old grafts of* G. biloba* were grown in an orchard at Yangtze University, China. For tissue expression of* GbWRKY2*,* GbWRKY16*, and* GbWRKY21*, the roots, stems, leaves, and male and female flowers of* G. biloba* grafts were collected, immediately frozen in liquid nitrogen, and kept at −80°C prior to total RNA extraction. The cultured callus lines, initiated from mature zygotic embryos of* G. biloba*, were maintained on liquid MS basal medium supplementing with 1.5 mg/L naphthaleneacetic acid (NAA) and 2 mg/L 6-benzyladenine (6-BA) on a rotary shaker at 100 rpm, in the light and at 25 ± 1°C. The suspension cultures were subcultured every 2 weeks and after four subcultures the differential was omitted. In the experiments for investigating induction by various elicitors, the callus lines were dipped into the appropriate treatment such as 100 *μ*mol L^−1^ MeJA, 100 *μ*mol L^−1^ ABA, 100 *μ*mol L^−1^ SA, 40 *μ*mol L^−1^ ETH, and 200 mmol L^−1^ sodium chloride (NaCl), respectively, using ginkgo callus without any treatment as control. The cold and heat treatments were performed by placing the callus lines in a 4°C and 40°C growth room and the control in a 25°C growth room. The callus samples were harvested 0, 3, 6, 12, 24, 48, and 72 h after treatment and immediately frozen in liquid nitrogen, followed by storage at −80°C until use.

### 2.2. RNA and DNA Extraction

Total RNA was extracted from different tissues and all the treatments using CTAB method [42]. Genomic DNA was extracted from fresh leaves of* G. biloba* grafts following the CTAB method described by Xu et al. [43]. The RNA and DNA quality and quantity were determined by agarose gel electrophoresis and spectrophotometer analysis before use.

### 2.3. RNA-Seq Library Construction for Illumina Sequencing and Identification of WRKY Transcription Factor Gene from* G. biloba* Transcriptome

The extracted RNA was cleaned up using RNase-free DNaseI (Dalian TaKaRa, China) and purified using oligo-dT-attached magnetic beads. The purified mRNAs were cleaved into small pieces (200~500 bp) by super sonication. Cleaved mRNAs were used as templates to construct RNA-Seq library according to the manufacture's protocol. The constructed libraries were purified by the AMPure beads and recovered from the low melting agarose (2%) at the length of about 300 base-pair by the Qiagen Nucleic acid purification kits (CA Qiagen, USA). Sequencing run was performed on Illumina HiSeq 2500 sequencer following the manufacturer's protocols.

To obtain the complete* G. biloba* WRKY gene family, the male and female flowers of* G. biloba* transcriptome sequences were queried for 72* A. thaliana* WRKY protein sequences using TBLASTN searching program. All of the collected* G. biloba* WRKY candidates were primarily analyzed using protein family database (Pfam) (http://pfam.xfam.org/) to confirm the presence of WRKY conserved domains on their protein structure.

### 2.4. Expression Analysis by Quantitative Real-Time PCR

Quantitative real-time PCR (qRT-PCR) was performed according to the instructions provided for the Applied Biosystems 7500 Real-Time PCR system (Foster City, CA, USA) and the SYBR Premix Ex Taq II (Dalian TaKaRa, China). The RNA samples were reversely transcribed using the PrimeScript RT-PCR kit (Dalian TaKaRa, China). Expression levels of WRKY genes in* G. biloba* were normalized using the glyceraldehyde-3-phosphate dehydrogenase (*GbGAPDH*, GenBank accession number L26924) as an internal reference gene. Based on the screened transcripts, three annotated WRKY transcripts (T2_Unigene_BMK.10348 named* GbWRKY2*, T1_Unigene_BMK.13300 named* GbWRKY16*, and T1_Unigene_BMK.18545 named* GbWRKY21*) were selected for expression analysis. Primers were designed using the Sequence Detection System software and shown in Table 1. qRT-PCR was performed with 20 *μ*L reaction consisting of 2x SYBR Premix Ex Ta 10 *μ*L, 0.4 *μ*L each primer and 50x ROX Reference Dye II, and 100 ng cDNA as the template. The thermal cycler conditions recommended by the manufacturer were used with first stage at 95°C for 30 s, and the second stage (40 cycles) at 95°C for 5 s followed by 60°C for 34 s, and the third stage at 95°C for 15 s followed by 60°C for 1 min and 95°C for 15 s. The amplification of the target genes was monitored every cycle by SYBR green fluorescence. The Ct (threshold cycle), defined as the real-time PCR cycle at which a statistically significant increase of reporter fluorescence was first detected, was used as a measure for the starting copy numbers of the target gene. Three replicates of each experiment were performed, and data were analysed by the Livak method [44] and expressed as normalized relative expression level (2^-ΔΔCT^) of the respective genes in various samples. In each case, three technical replicates were performed for each of at least three independent biological replicates.

### 2.5. Cloning of the Full-Length cDNA and Genomic DNA of* GbWRKY2*

Two specific primers WRKY2-FP and WRKY2-RP (Table 1) were designed and synthesized (Shanghai Sangon, China) based on the transcriptome data to obtain the internal fragment. One step RT-PCR was carried out and a fragment of 689 bp was obtained by using one step RT-PCR kit (Dalian TaKaRa, China) under the following program: 50°C for 30 min and 94°C for 3 min, followed by 35 cycles of amplification (94°C for 1 min, 54°C for 1 min, and 72°C for 1 min), and then followed by extension for 10 min at 72°C. The PCR product was purified and cloned into pMD18-T vector (Dalian TaKaRa, China) followed by sequencing. Subsequent BLAST results confirmed that amplified product was partial fragment of WRKY gene.

Based on the sequence of the internal fragment of* GbWRKY2* gene, the specific primers WRKY2-3/WRKY2-5 and the nested primer WRKY2-3N/WRKY2-5N (Table 1) were designed to amplify the 3′ and 5′ end of the* GbWRKY2* gene using the SMART RACE cDNA Amplification kit (Clontech, USA). The 3′-RACE-PCR and 5′-RACE-PCR were performed according to the manufacturer's instructions. The PCR products were purified and cloned into pMD18-T vector for sequencing. After comparing and aligning the sequence of 3′-RACE, 5′-RACE, and the internal region products, the full-length cDNA sequence of* GbWRKY2* was obtained and verified through PCR amplification using 3′-Ready cDNA as the template and a pair of primers WRKY2-G1 and WRKY2-G2 (Table 1) under the following conditions: 94°C for 3 min, followed by 35 cycles of amplification (94°C for 20 s, 56°C for 30 s, and 72°C for 2 min). After sequencing, the full-length cDNA of* GbWRKY2* was subsequently analyzed for molecular characterization. Two gene-specific primers, WRKY2-G1 and WRKY2-G2, designed based on the cDNA sequence were used to amplify the genomic sequence of* GbWRKY2*.

### 2.6. Amplification of the Promoter Region of* GbWRKY2*

Ginkgo genome walker libraries were constructed using the Genome Walker Universal Kit User (Clontech, USA). To clone the promoter region of* GbWRKY2*, two round PCR were performed using gene-specific primers (WRKY2-SP1 and WRKY2-SP2) that were designed according to the sequence of* GbWRKY2* cDNA and the adapter primers (AP1 and AP2) provided by the kit. Their sequences were shown in Table 1. After the nested PCR was carried out, amplified fragments were cloned and then sequenced. The sequences that extended upstream of the cDNA of* GbWRKY2* were isolated as the 5′-upstream region of* GbWRKY2* gene and used for further analysis.

### 2.7. Promoter Deletion-GUS Constructs and Agrobacterium-Mediated Transient Expression Assay

For construction of the* GbWKRY2* promoter-driven GUS fusion genes, the* GbWRKY2* promoter fragment-covering regions were amplified by PCR. Forward primers (Table 1) were designed to correspond to the −1363, −1018, −721, −668, −521, −288, −137, and −48 of* GbWRKY2* promoter and reverse primers, WRKY2P-anti and WRKY2-5UTR-R, were complementary to the 3′-end sequence of* GbWRKY2* promoter and 5′-UTR region, respectively. In addition, a supplementary fragment was amplified using primers WRKY2-1363 and WRKY2P-anti lacking the 5′-UTR region of* GbWRKY2*. Each fragment was digested with* Pst*I/*Bam*HI and subcloned into* Pst*I/*Bam*HI-digested pBI121 to generate seven promoter deletion derivatives. The promoterless construct (pBI121) was used as negative control. All constructs were verified by sequence analysis. Each promoter-GUS fusion construct was introduced into* A*.* tumefaciens* LBA4404 via electroporation.* Agrobacterium*-mediated transient expression assays were performed according to the method of An et al. [45]. Agrobacterium-mediated transient expression was conducted on fully expanded tobacco leaves, and the intercellular spaces of the intact leaves were infiltrated with the bacterial suspension. After agroinfiltration, tobacco plants were maintained in a moist chamber at 24°C for 48 h. The GUS activity was examined in the tobacco leaves coinfiltrated with* Agrobacterium* strains harboring different* GbWRKY2* promoter-GUS fusion constructs.

### 2.8. GUS Activity Assay

The seedlings of tobacco transformant were ground in liquid nitrogen. The crude proteins were extracted with 50 mM phosphate buffer, pH 7.0, containing 10 mM *β*-mercaptoethanol, 10 mM EDTA, and 0.1% Trition X-100. Homogenates were cleared by centrifugation, and the supernatants were assayed by Bradford assay for total protein (DU700, Beckman, Germany). GUS activity was measured as described by Jefferson et al. [46].

### 2.9. Southern Blot Analysis

Aliquots of DNA (30 *μ*g/sample) were digested overnight at 37°C with* Bam*HI and* Pst*I, respectively, which does not cut within the probe region, fractionated by 0.85% agarose gel electrophoresis and transferred to a Hybond-N^+^ nylon membrane (Amersham Pharmacia, UK). The 283-bp probe was generated by PCR using genomic sequence of* GbWRKY2* including intron as template with primers FWR2PIN and RWR2PIN (Table 1). Probe labeling (biotin), hybridization, and signal detection were performed using Gene Images random prime labeling module and CDP-Star detection module following the manufacturer's instructions (Amersham Pharmacia, UK). The film was washed under stringent conditions (60°C) and signals were visualized by exposure to Fuji X-ray film at room temperature for 1.5 h.

### 2.10. Bioinformatic Analysis and Molecular Evolution Analyses

The obtained sequences were analyzed using bioinformatic tools at websites (http://www.ncbi.nlm.nih.gov and http://www.expasy.org/). The software vector NTI Suite 10 was used for sequence multialignment. The isolated 5′-upstream sequence was analyzed for the putative* cis*-acting regulatory elements using the PLACE (http://www.dna.affrc.go.jp/PLACE/) and the Signal Scan Program PlantCARE (http://bioinformatics.psb.ugent.be/webtools/plantcare/html/) database. Phylogenetic tree analysis of* GbWRKY2* and known WRKYs from other plant species retrieved from GenBank were aligned with CLUSTAL W. Subsequently, a phylogenetic tree was constructed by neighbor-joining (NJ) method. The reliability of the tree was measured by bootstrap analysis with 1000 replicates.

## 3. Results

### 3.1. *WRKY* Candidate Genes Were Identified in* G. biloba* Transcriptome

Transcriptomes of male and female flowers from* G. biloba* were sequenced using Illumina RNA-seq technology, yielding 20 and 19 million transcript reads, respectively. Using the PFAM protein family database with the WRKY domain (PF03106), we identified a total of 28 WRKY transcription factors (Table 2). There were differences in the lengths of proposed sequences of 28* WRKY* genes, ranging from 258 bp to 2325 bp. These differences may have been due largely to assembly error in partial chromosomal regions and require further confirmation. Gene expression level was estimated using RPKM (reads per kilobase per million mapped reads) value. Among these 28 WRKY transcripts, 20 WRKY genes were identified to be highly expressed in male flowers and the others were highly expressed in female flowers. Subsequently, we selected* GbWRKY2*,* GbWRKY16*, and* GbWRKY21* for expression analysis.

### 3.2. Expression Analysis in Different Tissues

**Figure 1:**
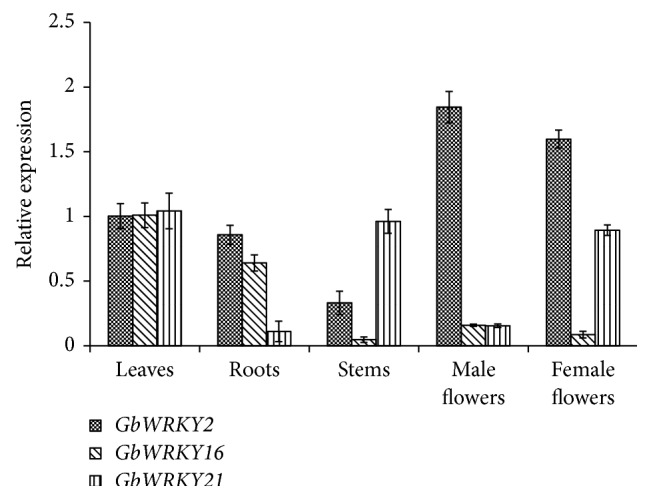
The expression levels of* GbWRKY2*,* GbWRKY16*, and* GbWRKY21* in* Ginkgo biloba* different tissue. Relative expression levels of* GbWRKY2*,* GbWRKY16*, and* GbWRKY21* in different tissues with* GbGAPDH* gene as internal reference gene. Total RNA samples were isolated from leaves, roots, stems, male flowers, and female flowers, respectively. Each sample was individually assayed in triplicate. Values shown represent the mean reading from three plants and the error bars indicated the standard errors of the mean.

The expression profile of* GbWRKY2*,* GbWRKY16*, and* GbWRKY21* was assessed by qRT-PCR in leaves, roots, stems, and male and female flowers of 14-year-old graft ginkgo trees (Figure 1). The results showed that all of three genes transcripts could be detected in all tissues, but at different expression levels. The highest expression level of* GbWRKY2* was observed in flowers and significantly higher than in the leaves and roots. The lowest expression level of* GbWRKY2* was observed in stems and was significantly lower (*P* < 0.05) in other tissues. In contrast, the expression level of* GbWRKY16* was very low in flowers, and the expression level of* GbWRKY21* was significantly higher in leaves, stems, and female flowers than in roots and male flowers.

### 3.3. Effect of Abiotic Stresses on the Expression of Three* WRKY* Genes

**Figure 2:**
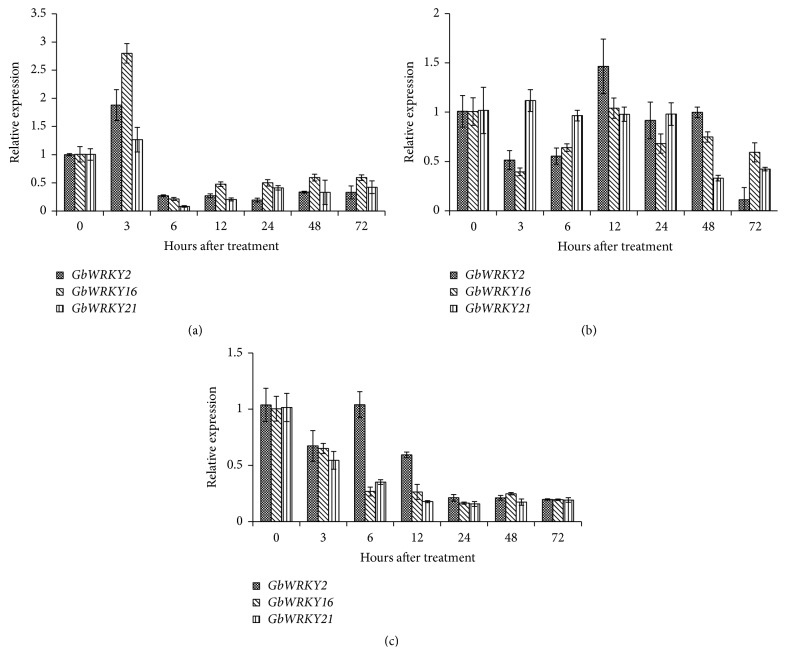
Relative expression levels of* GbWRKY2*,* GbWRKY16*, and* GbWRKY21* under salinity (a), cold (b), and heat (c) treatments. Relative expression of* GbWRKY2*,* GbWRKY16*, and* GbWRKY21* at various hours after treatment under salinity, cold, and heat treatments with* GbGAPDH* gene as internal reference gene. Each sample was individually assayed in triplicate. Values shown represent the mean reading from three treated samples and the error bars indicated the standard errors of the mean.

To determine the function of* GbWRKY2*,* GbWRKY16*, and* GbWRKY21* in response to abiotic stress, we investigated the time-course expression patterns of these three genes in gingko callus treated with NaCl, cold, and heat. As shown in Figure 2(a),* GbWRKY2* and* GbWRKY16* transcript levels were enhanced by NaCl, showing a 1.9-fold and 2.8-fold, respectively, compared with the control at 3 hpt (hours after treatment). Afterwards, the transcript levels of these two genes were decreased sharply after 6 hpt.* GbWRKY21* transcript level was increased slightly and then decreased along with the treatment.* GbWRKY2* transcript levels were also somewhat enhanced by low temperature (4°C) at 12 hpt (Figure 2(b)) but were decreased slightly along with the treatment time under high temperature (40°C) stress (Figure 2(c)). The relative expressions of* WRKY16* and* GbWRKY21* were significantly decreased under both low and high temperature treatments (Figure 2(c)).

### 3.4. Effect of Hormones on the Expression of Three* WRKY* Genes

**Figure 3:**
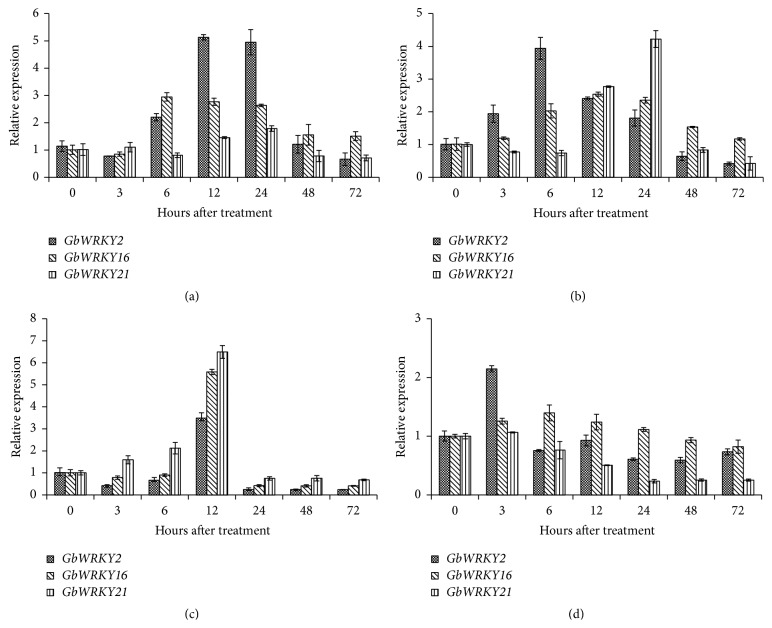
Relative expression levels of* GbWRKY2*,* GbWRKY16*, and* GbWRKY21* with ABA (a), SA (b), ETH (c), and MeJA (d) treatments. Relative expressions of* GbWRKY2*,* GbWRKY16*, and* GbWRKY21* at various hours after treatment under ABA, SA, ETH, and MeJA treatments with* GbGAPDH* gene as internal reference gene. Each sample was individually assayed in triplicate. Values shown represent the mean reading from three treated samples and the error bars indicated the standard errors of the mean.

Phytohormones, such as SA, MeJA, ABA, and ETH, serve as important signaling molecules and play crucial roles in controlling the expression of downstream defense genes and physiological reactions against biotic and abiotic stresses. To assess the possible involvement of three* WRKY* genes in signaling pathways utilized by these hormones, the transcript level of* GbWRKY2*,* GbWRKY16*, and* GbWRKY21* in ginkgo callus was determined by qRT-PCR treated with ABA, SA, ETH, and MeJA. All the transcript levels of the* GbWRKY2*,* GbWRKY16*, and* GbWRKY21* were significantly (*P* < 0.05) increasingly treated with ABA compared with the control. The strongest response of* GbWRKY2* (5.1-fold compared with the control),* GbWRKY16* (2.9-fold compared with the control), and* GbWRKY21* (1.8-fold compared with the control) to ABA treatment was observed at 12, 6, and 24 hpt, respectively (Figure 3(a)).

In regard to SA,* GbWRKY2* transcript level was increased between 3 and 24 hpt (Figure 3(b)). The highest levels of* GbWRKY2* transcript level (3.9-fold compared with the control) were reached at 6 hpt.* GbWRKY16* transcript level was sustainably upregulated during SA treatment and the highest level of* GbWRKY16* (2.5-fold compared with the control) was reached at 12 hpt. In contrast,* GbWRKY21* transcript level was firstly decreased after treatment and increased between 12 and 24 hpt, treated with SA compared with the control. The strongest response to SA (4.2-fold compared with the control) was observed at 24 hpt.

Figure 3(c) showed that the transcript levels of* GbWRKY2* and* GbWRKY16* were decreased sharply before 12 hpt but enhanced rapidly and reached the peak, nearly 3.5-fold and 5.6-fold compared with the control, respectively, under ETH treatment.* GbWRKY21* transcript level was continuously upregulated until 12 hpt reaching a 6.5-fold compared with the control and then was decreased.

For MeJA treatment,* GbWRKY2* transcript level was significantly (*P* < 0.05) induced between 0 and 3 hpt, after which they decreased to the similar level to the control (Figure 3(d)). In contrast to GbWRKY2, no significant effect of MeJA was observed in* GbWRKY16* transcript level, while* GbWRKY21* transcript level was significantly (*P* < 0.05) decreased by MeJA.

### 3.5. Cloning of* GbWRKY2* and Its Sequence Analysis

The WRKY genes have previously been determined to respond to MeJA and are involved in many life processes, including stress resistance [47], secondary metabolism [23], and plant defense [48]. The exploration of the function of MeJA-inducible WRKY genes in* G. biloba* would be beneficial for discovering genes involved in stress resistance and in secondary metabolite biosynthesis in* G. biloba.* Since qRT-PCR analysis indicated that* GbWRKY2* might be involved in the signal transduction of MeJA in stress resistance of ginkgo, we cloned the full-length cDNA, genomic DNA, and promoter region of* GbWRKY2* to further study the function of* GbWRKY2* in stress defense.

**Figure 4:**
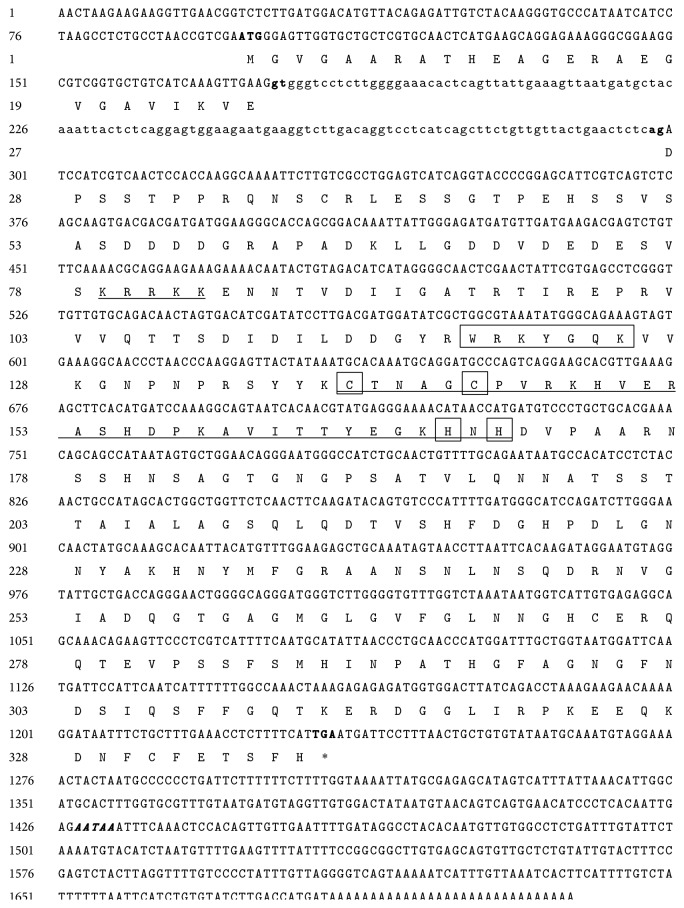
The full-length cDNA, intron, and deduced amino acid sequence of* GbWRKY2* gene. The exons sequence is indicated in capital letter and the intron is indicated in lowercase. The start codon (ATG), the stop (TAG), and putative exon-intron splicing sites (gt/ag) are shown by bold letters. One putative polyadenylation signal is bold and italic. A putative nuclear localization signal is underlined. The WRKY domain and the C and H residues in the zinc finger motif (CX_4_CX_23_HX_1_H) are boxed. The zinc finger motif (CX_4_CX_23_HX_1_H) is marked by underline.

The full-length cDNA of* GbWRKY2* (GenBank accession number KP987204) was 1,713 bp and contained a 1,014 bp open reading frame encoding a 337 amino acid proteins. One possible polyadenylation signal (AATAA) was found at 194 bp position downstream from the stop codon (Figure 4). A pair of specific primers was designed to synthesize cDNA between the start codon and the terminator codon of* GbWRKY2*. The genomic sequence of* GbWRKY2* (GenBank accession number KP987205) with a length of 1,137 bp was amplified and exhibited 100% identity of the coding region of the full-length cDNA sequence. The genomic sequence of* GbWRKY2* contained one intron with a length of 123 bp. This intron was smaller than that of* Arabidopsis* (241 bp) and rice (868 bp). The putative splicing site obeyed the GT/AG rule [11] (Figure 4). Compared with the identities of the nucleotides of other plants in NCBI, the identities of* GbWRKY2* with the nucleotide sequence of the WRKY gene from* Picea glauca*,* P. sitchensis*,* Amborella trichopoda*,* Medicago truncatula*, and* Morus notabilis* were 83%, 82%, 81%, 81%, and 83%, respectively.

### 3.6. Southern Blot Analysis

**Figure 5:**
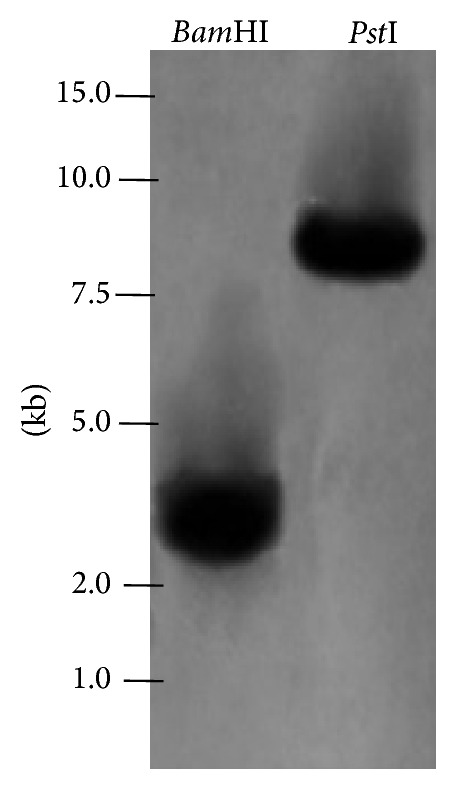
Southern blot analysis of* GbWRKY2*. Genomic DNA of* G. biloba* was digested with* Bam*HI and* Pst*I, separated on a 0.85% agarose gel, blotted onto a positively charged nylon membrane and probed with a biotin-labeled* GbWRKY2* fragment.

To investigate the genomic organization of* GbWRKY2*, we carried out a Southern blot analysis by digesting genomic DNA of* G*.* biloba* with* Bam*HI and* Pst*I, followed by hybridization with a 283 bp probe generated by PCR using the genomic sequence of* GbWRKY2* as template. As shown in Figure 5, only one hybridized band was detected in each lane, indicating that* GbWRKY2* was a single copy gene in* G. biloba*.

### 3.7. Analysis of* GbWRKY2* Protein

**Figure 6:**
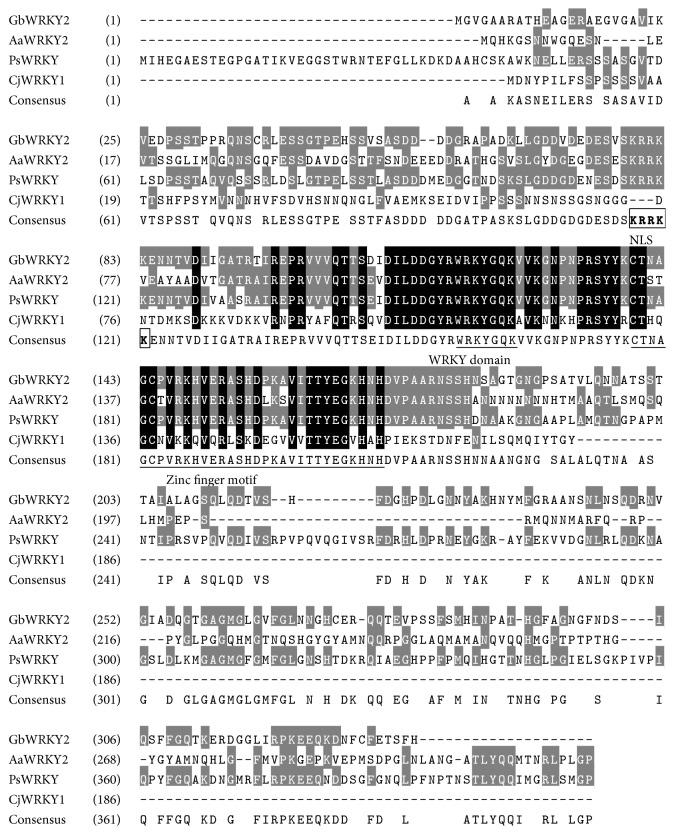
Sequence multialignment of the deduced GbWRKY2 protein with other WRKYs. The completely identical amino acids are indicated with white foreground and black background. The conserved amino acids are indicated with white foreground and grey background. Nonsimilar amino acids are indicated with black foreground and white background. The WRKY domain and zinc finger motif (CX_4_CX_23_HX_1_H) are underlined. A putative nuclear localization signal (KRRKK) is shown by bold letters and boxed. The GenBank accession numbers of WRKY proteins and translation of their names are shown as follows: GbWRKY2:* Ginkgo biloba*; CjWRKY1:* Coptis japonica var. dissecta* BAF41990.1; PsWRKY:* Picea sitchensis* ADE77055.1; AaWRKY2:* Artemisia annua* AGR40498.1.

The* GbWRKY2* was predicted to encode a protein of 337 amino acid residues. The relative molecular mass and theoretical isoelectric point (pI) of the predicted protein were 36.25 kDa and 6.16, respectively. BLASTP analysis in NCBI revealed that the deduced GbWRKY2 amino acid showed high identity to known WRKY TFs from other plants (Figure 6). The analysis of the deduced amino acid revealed that GbWRKY2 contained one conserved WRKY domain and a zinc finger motif of C_2_H_2_ (Figure 6), suggesting GbWRKY2 is a member of the WRKY II family [10, 11]. Like other members of WRKY II family, the conserved domain zinc finger motif for binding Zn^+2^ ion required for protein function presents at similar positions in GbWRKY2. In addition, sequence analysis using WoLF PSORT (http://www.genscript.com/psort/wolf_psort.html) indicated that the predicted GbWRKY2 protein contains a putative nuclear localization signal (^78^KRRKK^82^). All the observed conservations of these domains and motifs in all aligned sequences, especially in* G*.* biloba*, suggested the function of the GbWRKY2 protein.

**Figure 7:**
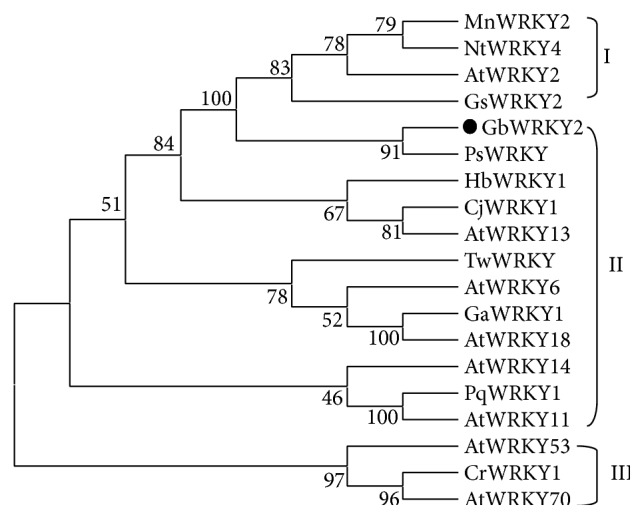
Phylogenetic tree of the sequences of GbWRKY2 and other plants WRKY protein. The numbers at each node represented the bootstrap values (with 1000 replicates). The GenBank accession numbers of WRKY proteins and translation of their names are shown as follows: GbWRKY2:* Ginkgo biloba*; MnWRKY2:* Morus notabilis* XP_010092241.1; NtWRKY4:* Nicotiana tabacum* BAA86031.1; AtWRKY2:* Arabidopsis thaliana* AED96743.1; GsWRKY2:* Glycine soja* KHN40472.1; PsWRKY:* Picea sitchensis* ADE77055.1; HbWRKY1:* Hevea brasiliensis* ADF45433.1; CjWRKY1:* Coptis japonica var. dissecta* BAF41990.1; AtWRKY13:* Arabidopsis thaliana* AEE87071.1; TwWRKY:* Taxus wallichiana var. chinensis* AEW91476.1; AtWRKY6:* Arabidopsis thaliana* AEE33948.1; GaWRKY1:* Gossypium arboreum* AAR98818.1; AtWRKY18:* Arabidopsis thaliana* AEE85961.1; AtWRKY14:* Arabidopsis thaliana* AEE31256.1; PqWRKY1:* Panax quinquefolius* AEQ29014.1; AtWRKY11:* Arabidopsis thaliana* AEE85928.1; AtWRKY53:* Arabidopsis thaliana* AEE84809.1; CrWRKY1:* Catharanthus roseus* HQ646368; AtWRKY70:* Arabidopsis thaliana* AEE79517.1.

To investigate the evolutionary relationships among GbWRKY2 and other WRKY TF proteins, the phylogenetic tree was constructed using neighbor-joining method (Figure 7). The results showed a total of 19 WRKY proteins divided into three classes. Among these proteins, MnWRKY2, NtWRKY4, GsWRKY2, and AtWRKY2 belonged to class I; AtWRKY53, AtWRKY70, and CrWRKY1 belonged to class III. GbWRKY2, HbWRKY1, CjWRKY1, TwWRKY, and GaWRKY1, as well as PqWTKY1, clustered in Group II. Furthermore, GbWRKY2 and PsWRKY of gymnospermae clustered in the same branch, implying that these two WRKY TFs may exhibit a close genetic relationship and display similar protein function.

### 3.8. Analysis of 5′-Flanking Sequence of* GbWRKY2*

Based on the DNA sequence of* GbWRKY2* gene, the nested primer is designed, and the 5′-flanking sequence (KP987205) with 1,363 bp of* GbWRKY2* was isolated by genome walking method. Taking into account the result of 5-RACE PCR, we concluded the initiation site of* GbWRKY2* transcription at −97 bp in the upstream of ATG (Figure 1S in the Supplementary Material available online at http://dx.doi.org/10.1155/2015/607185). The promoter region of* GbWRKY2* possessed a typically high A + T content of 58.0%, which was commonly found in other plant promoters. The promoter region of* GbWRKY2* was then submitted to PLACE and PlantCARE databases for the analysis of putative* cis*-element. The result showed that the TATA box was located at −26 bp in the upstream of the transcription initiation site. Another conserved eukaryotic* cis*-element, the CAAT box, was also found at 232, 787, and 824 bp (Figure 1S), consistent with typical characteristics of the plant promoter. Other stress-related* cis*-elements were also observed in the promoter region of* GbWRKY2* (Table 3). For example, one low temperature response (CRTDREHVCBF2) [30], two drought stress response and aging response (ACGTATERD1) [27], and four disease defense (BIHD1OS) [28]* cis*-elements were found. Motifs related to light regulation, including three GT1 [49] and one IBOX [36], were also detected. Furthermore,* cis*-acting elements related to hormones were also predicted (Figure 1S). For example, we found one core motif for binding DPBF TF, three GT1 motifs, seven ARR1AT TF binding sites, and four PYRIMIDINE-boxes that participate in the signal response of ABA, salicylic acid, cytokinin, and gibberellin [26, 32, 39, 49], respectively, in the promoter region of* GbWRKY2*. Two ABA response* cis*-elements were also found at 10 and 585 bp position [24, 25]. Moreover, some TF binding sites were also detected. For example, five Dof TF binding sites were found in the promoter region of* GbWRKY2*, and these motifs participate in the signal response of auxin [50], jasmonic acid, or ethylene [51] in other plants. In addition, we found ten E-boxes, five MYB-boxes, nine MYC-boxes, and one W-box in* GbWRKY2* promoter, which is conservative binding motif of bHLH [33], MYB [38], MYC [37], and WRKY [12] TFs, respectively. Interestingly, the presence of a putative W-box binding site within the* GbWRKY2* promoter might indicate that this ginkgo gene can be subjected to autoregulation or can be modulated by other WRKY members [52, 53]. Prediction results showed that the regulatory* cis*-elements of* GbWRKY2* promoter were related to stress-induced response, indicating that the promoter may play an important role in response to external environmental stress and biological defense process.

### 3.9. Deletion Analysis of the* GbWRKY2* Promoter in Tobacco Leaf Tissues

**Figure 8:**
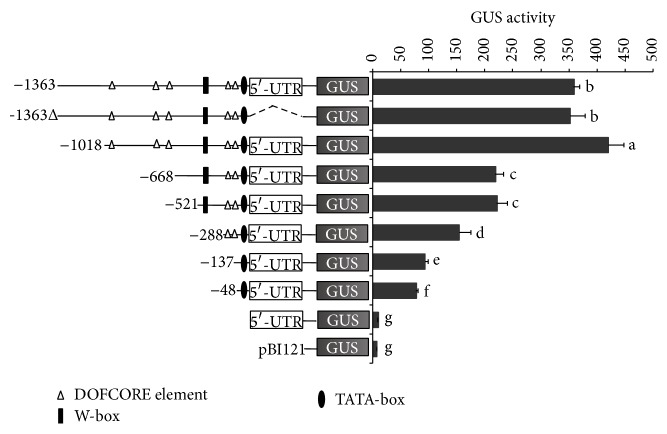
Deletion analysis of the GbWRKY2 promoter-driven GUS activity. Schematic diagram of the constructs used for GUS activity assays in leaves of transgenic tobacco plants is shown at left. Dash line pinpoints the deletion of the 5′-UTR region for the construct −1363Δ. Quantitative analyses of GUS activity of transgenic plants driven by deletion constructs of the* GbWRKY2* promoter are shown at right. Error bars represent standard deviation (SD). Data are mean ± SD from triplicate experiments. Values with different letters show significant differences at *P* = 0.05 according to the Fisher's least significant difference (LSD) test.

To gain insight into the functional role of the* GbWRKY2* promoter region, series deletions were sequential of the* cis*-elements and fusion of the remaining promoter to the GUS reporter gene was constructed. A promoterless construct (pBI121) was used as a negative control (Figure 8). Sequential 5′-deletions of the promoter were performed by PCR, and the responsiveness of the deleted versions of the* GbWRKY2* promoter was analyzed by transient assays in tobacco leaves. As shown in Figure 8, the GUS activity of the maximum-length containing 5′-UTR of* GbWRKY2* (construct −1363) was 358.36 nM 4-methylumbelliferone (MU) mg^−1^ protein min^−1^, although the GUS activity (352.19 nM 4-mg^−1^ protein min^−1^) of 5′-UTR deletion of full-length promoter (−1363Δ) was slightly less than that of construct −1363 but did not reach significant level (*P* > 0.05), suggesting that 5′-UTR had no effect on the transcript level of* GbWRKY2* promoter under our experimental condition. The highest level of GUS activity was observed in transgenic tobacco leaves harboring the −1018 promoter construct and significantly higher (*P* < 0.05) than that of the full-length promoter (construct −1363), implying that negative* cis*-elements may be present in the promoter region between −1363 and −1018. By contrast, deletion of the GbWRKY2 promoter from −1018 to −668 caused a significant (*P* < 0.05) reduction of the promoter expression. Successive deletion from −668 to −521 had no additional significant effect on GUS activity while further deletion to −228 containing a W-box causes a significant (*P* < 0.05) decrease of GUS activity. Likewise, both further deletions from −228 to −137 and from −137 to −48 led to 39.3% and 16.7% decrease of promoter expression, respectively. Finally, both the constructs containing 5′-UTR and negative constructs present a quasicomplete disappearance of GUS activity, suggesting 5′-UTR region had no contribution to GbWRKY2 expression.

## 4. Discussion

Given the important roles that WRKY transcription factors play in response to various stresses, the traditional gene-by-gene research was not a high-efficiency method to study the plant that had no genome sequence. High throughput sequencing data have been used for functional gene mining and have proven to be an effective method for metabolic gene discovery and others [23]. In this study, the transcriptome dataset was searched for WRKY transcription factors in* G. biloba* and a total of 28 candidate WRKY genes were identified. Of these WRKY genes, we selected* GbWRKY2*,* GbWRKY16*, and* GbWRKY21* to expression analysis. Interestingly, our data showed that the transcript level of* GbWRKY2* was predominantly observed in ginkgo inflorescence and strongly induced by MeJA, implying* GbWRKY2* might play dual roles in the development of ginkgo inflorescence and defense responses by mediating MeJA signaling.

### 4.1. *GbWRKY2* Is a Group II WRKY Transcription Factor

A ginkgo gene (*GbWRKY2*) encoding a protein with sequence homology to members of the WRKY family has been characterized. This newly characterized gene is the first WRKY factor described in* G. biloba* and presents structural hallmarks that allow us to classify it within Group II of WRKY TFs [11]. The phylogenetic analysis also clearly showed that* GbWRKY2* belong to Group II WRKY family. Numerous members of plant WRKY genes belong to Group II family and play roles in transcriptional reprogramming associated with resistance to various stresses [11]. A predicted nuclear localization signal (NLS) (Figure 4) strongly suggests that GbWRKY2 protein is translocated into the nucleus to control gene expression. These data suggested that* GbWRKY2* might play an important role in defense response of ginkgo to biotic and abiotic stresses.

### 4.2. Multiple cis-acting Elements Response to Stress and Hormone in the Promoter Region of* GbWRKY2*

Stress-inducible gene expression is transcriptionally regulated by a change in the level and/or activity of sequence-specific DNA-binding transcription factors bound to specific* cis*-acting elements of promoter regions [54]. These regulatory factors are involved in the activation, suppression, and modulation of various signaling pathways in plant cells on biotic and abiotic stresses. Many plant promoters have therefore been identified and isolated, and genetic engineering in plants has been greatly enhanced using individual promoters [55]. Thus, we isolated a 1,363 bp-length promoter of* GbWRKY2* (Figure 1S). Bioinformatics analysis showed that multiple* cis*-acting elements including fundamental and special elements associated with abiotic stresses and hormone regulations were found in the* GbWRKY2* promoter (Table 3), indicating that* GbWRKY2* gene might respond to various environment stimulus. Furthermore, a series of 5′ end deletion fragments of this promoter were detected using transient expression assays, which demonstrated that the activity of a truncated promoter from position −1 to −48 bp retained the ability to initiate the expression of* GUS* in transgenic tobacco leaves. This region is enough to keep the basic function of* GbWRKY2* promoter. Bioinformatics analysis showed a TATA box was located in the region between −1 and −48 bp. Together taken into the results of bioinformatics and deletion analysis, we conclude that the region of −1 to −48 bp is the equal of core promoter and positions of TATA box correspond to the typical core promoter model [56]. Moreover, our experiments showed that deletion of the promoter regions between positions −1018 and −668 or between positions −288 and −137 caused very significant (*P* < 0.01) reductions in promoter activity suggesting that these regions likely contain potential transcription-enhancing* cis*-acting elements. Sequence analysis discussed above indicates that both regions contain three and two Dof TF binding sites, respectively, which may be important for the hormonal responsiveness of* GbWRKY2* promoter.

### 4.3. *GbWRKY2* Might Participate in the Development of Ginkgo Inflorescence

Similar to expression patterns observed in other plant species, three* GbWRKYs* were found to be expressed in all tissues we used, but at different expression levels. Moreover, the transcription of* GbWRKY2* was observed abundant in flowers. In contrast, expression levels of* GbWRKY16* were very low in flowers. And the expression levels of* GbWRKY21* were significantly higher in leaves, stems, and female flowers than in roots and male flowers. The result may indicate that* GbWRKY2*,* GbWRKY16*, and* GbWRKY21* played different roles involved in regulating plant developmental and physiological processes. The highest expression level of* GbWRKY2* is in flowers while the lowest expression level of in stems, consistent with the spatial expression profile of* ChWRKY2* from* Corylus heterophylla* [57]. Given highest transcript level of* GbWRKY2* in flowers, it can be speculated that* GbWRKY2* might play an important role in tolerance to low temperature in inflorescence, which developed in cold early spring. Thus, we deduced that* GbWKY2* likely exhibited similar function of* ChWRKY2* TF to participate in the developmental process of ginkgo inflorescence.

### 4.4. *GbWRKYs* Might Play Role in Stress-Related Signal Pathways

Salinity is an important abiotic stress factor, usually affecting plant growth, development, survival, and crop productivity. Thus, understanding the complex mechanism of salinity tolerance is important for agriculture production. Interestingly, several WRKY proteins were shown to be involved in plant salinity stress response. For example, the expression of* CmWRKY17* was induced by salinity in* Chrysanthemum morifolium* and overexpression of* CmWRKY17* in Chrysanthemum and Arabidopsis increased the sensitivity to salinity stress [58]. The transcripts of two closely related WRKY TFs (*AtWRKY25* and* AtWRKY33*) from Arabidopsis were increased by NaCl treatment and both the Atwrky33 null mutants and Atwrky25Atwrky33 double mutants showed moderately increased NaCl sensitivity [59]. Poplar species increase expressions of transcription factors to deal with salt environments. Salinity increased heat-shock transcription factor (HSF) transcription in* P. euphratica* and* PeHSF* binds the cis-acting heat shock element (SHE) of the* PeWRKY1* promoter, thus activating* PeWRKY1* expression [60]. Similarly, the first evidence pointing towards a role for* GbWRKY2* and* GbWRKY16* in defense comes from the data showing that the* GbWRKY2* and* GbWRKY16* transcript is dramatically upregulated in ginkgo in response to NaCl. Further study on suppression/overexpression of* GbWRKY2* and* GbWRKY16* was required for unveiling the molecular mechanism of* GbWRKY2* and* GbWRKY16* gene participation in ginkgo tolerance to salt.

Temperature that exceeds an organism's optimal tolerance range is considered as an important abiotic stress factor. Tremendous work has been done in the past two decades to reveal the complex molecular mechanism in plants' responses to extreme temperature and there is increasing evidence that WRKY proteins are involved in responses to both heat and cold stresses. For example, a WRKY TF in* Nicotiana tabacum* responds to a combination of drought and heat stress [61]. Another example is that transgenic Arabidopsis plants overexpressing* GmWRKY21* exhibited increased tolerance to cold stress when compared with wild-type plants [62]. In our studies, the expression levels of* GbWRKY2*,* GbWRKY16*, and* GbWRKY21* were all repressed by heat stress. The expression level of* GbWRKY2* was upregulated by cold stress and there was no significant effect of cold stress on* GbWRKY16* transcript levels. In contrast, the expression level of* GbWRKY21* was downregulated by cold stress. We also reported for the first time an increase of* GbWRKY2* transcript level by cold but decrease by heat, consistent with the observation of cold responsive* cis*-elements (CRTDREHVCBF2 and MYC recognition site) (Table 3). Although low-temperature related WRKYs were isolated in several species [62–64]. The mechanism of how WRKYs respond to cold signals and regulated the expression downstream genes is still largely unknown. Further work is needed to elucidate the function of these important genes in low-temperature related signal pathways.

ABA is an important signal molecule related to abiotic stress. Previous studies had demonstrated that WRKY proteins may act as activator in ABA signaling [65]. It was reported that the expression of* LtWRKY21* in* Larrea tridentata* was shown to function as an activator to control ABA-regulated expression of genes [66]. Recently, Sun et al. also [67] reported that 13 numbers of WRKY family in* O. sativa* were upregulated by ABA, indicating that these* OsWRKY* genes may play an important role in the response to abiotic stress, particularly as a key regulatory factor in ABA signaling pathway. MYBCORE, DPBF, and ABRE are known to involve in ABA and drought responses. Some MYBCORE, DPBF, and ABRE motifs were detected in the* GbWRKY2* promoter by bioinformatic analysis (Figure 1S). The upregulated expression of* GbWRKY2*,* GbWRKY16*, and* GbWRKY21* in ginkgo callus by ABA gave the direct evidence, suggesting that these WRKY genes probably function as key components during ABA signaling. Our results also showed that all three* GbWRKYs* were upregulated by ABA, indicating that these* GbWRKY* genes may participate in ABA signaling pathway responding to abiotic stress.

The upregulation of* GbWRKY2* by SA is expected because three* cis-*elements (GT1 motifs) associated with SA were identified in the* GbWRKY2* promoter region. Interestingly,* GbWRKY2* also responds with a strong induction to a wide range of molecules such as MeJA and ETH, which constitute key signaling elements modulating defense responses to pathogens. This suggests that* GbWRKY2* is a common component in the signaling pathways mediated by these hormones. Numerous studies report the induction of WRKY gene expression in response to SA and MeJA [13, 68, 69], but few analyses report the effect of other signal molecules such as ETH [38, 70]. Pathways involving MeJA and ET are considered to be mainly effective against necrotrophic pathogens, insects, and wounding, whereas those involving SA are more effective against biotrophs [71]. Thus, little attack of pathogens and insects in ginkgo may be due to the fact that WRKY protein, such as* GbWRKY2*, played important roles in defense responses by mediating SA, MeJA, and ETH signaling.

Taken together,* GbWRKY2*,* GbWRKY16*, and* GbWRKY21* were activated by more than one type of stress condition.* GbWRKY2* was observed to be upregulated in response to many different sources of stress, including salinity, ABA, SA, ETH, and MeJA treatment.* GbWRKY16* was observed to be upregulated in response to salinity, ABA, SA, and ETH treatment.* GbWRKY21* was observed to be upregulated in response to ABA, SA, and ETH treatment.* GbWRKYs* were upregulated in response to more than two types of stress which supported the occurrence of cross-talk between signal transduction pathways in response to different stress conditions in plants. Moreover,* GbWRKYs* displayed complex expression patterns in response to the stress. For example,* GbWRKY2* and* GbWRKY16* were upregulated at 3 hpt in response to salinity treatment and then downregulated quickly, demonstrating that* GbWRKYs* could be quickly and instantaneously responded to the stress.* GbWRKY16* was not disturbed by the cold and MeJA treatment.* GbWRKY16* was sustainably upregulated during the whole SA treatment. All of these showed that* GbWRKYs* played complicated and essential roles in defense in response to the stress.

## 5. Conclusion

This study presents the isolation and expression profile of the novel WRKY transcription factor gene* GbWRKY2*, which encodes a protein of 337 amino acid residues. The protein domain and phylogenetic analysis also showed that* GbWRKY2* belong to Group II WRKY family. Bioinformatics analysis showed that multiple* cis*-acting elements including fundamental and special elements associated with abiotic stresses and hormone regulations were found in the* GbWRKY2* promoter. Expression pattern analysis suggested that* GbWRKY2* might play an important role in tolerance to salt and cold stresses and defense responses by mediating ABA, SA, MeJA, and ETH signaling. Studies on downstream function genes regulated by WRKY TF and mutual regulation between WRKY TFs in* G. biloba* have not been reported. On the basis of response abiotic adversity of* GbWRKY2*, we establish the binary overexpression vector of this gene. Studies on the genetic transformation of this gene in the callus of* G. biloba* are underway. This study will provide a basis to further reveal that the upregulated gene expression can strengthen the ability of plants to resist abiotic stress and identify some targeted genes regulated by WRKY TF in* G. biloba*. The present work on cloning and characterization of* GbWRKY2* provided new clues for future studies on Ginkgo response to various stresses, such as sanity, drought, cold, and disease.

## Supplementary Material

Figure S1. Nucleotide sequence of the 5'-upstream sequence of GbWRKY2. Various cis-elements were predicted from PlantCARE and PALCE plantforms. Coding region for GbWRKY2 gene is underlined, and translation start site is indicated by a translated methionine under ATG and shadowed. The transcription start site (TSS) and TATA-box are indicated as +1 and −26, respectively. Description of various cis-elements highlighted in the sequence is tabulated in Table 3.

## Figures and Tables

**Table 1:** The primers used in this study.

Primers	Sequence (5′-3′)	Description
WRKY2-FP	CCCATAATCATCCTAAGCCTCTG	Forward primer for *GbWRKY2* cDNA
WRKY2-RP	ATGGGACACTGTATCTTGAAGTTGAG	Reverse primer for *GbWRKY2* cDNA
WRKY2-3	AGAAAGTAGTGAAAGGCAACCCTAACCCAA	Forward primer for 3′-RACE, outer
WRKY2-3N	CCATAGCACTGGCTGGTTCTCAACTTCAAG	Forward primer for 3′-RACE, nested
WRKY2-5	CAACTTTGATGACAGCACCGACGCC	Forward primer for 5′-RACE, outer
WRKY2-5N	CGGTTAGGCAGAGGCTTAGGATGATTA	Forward primer for 5′-RACE, nested
WRKY2-G1	ATGGGAGTTGGTGCTGCT	Forward primer for *GbWRKY2* gDNA
WRKY2-G2	TCAATGAAAAGAGGTTTCAAAGC	Reverse primer for *GbWRKY2* gDNA
WRKY2-SP1	CCCTTCCATCATCGTCGTCACTTGC	Reverse primer for 5′-promoter, outer
WRKY2-SP2	ACGCCTTCCGCCCTTTCTCCTGCTT	Reverse primer for 5′-promoter, nested
WRKY2-U	AGTGCCAGCAATCAGCGTT	Gene forward primer for qRT-PCR
WRKY2-D	AGCCCTTGATGGGTTTCTGT	Gene reverse primer for qRT-PCR
WRKY16-U	ATCAGTCCACAAGCAGACCTCA	Gene forward primer for qRT-PCR
WRKY16-D	TTGTCATTAGCAACACTGCCATC	Gene reverse primer for qRT-PCR
WRKY21-U	GAATAGTATGGGTTCCAGTAGGTCG	Gene forward primer for qRT-PCR
WRKY21-D	AACCTTTGATAGGCTTCTGTCCA	Gene reverse primer for qRT-PCR
GAPDH-U	GGTGCCAAAAAGGTGGTCAT	Gene forward primer for qRT-PCR
GAPDH-D	CAACAACGAACATGGGAGCAT	Gene reverse primer for qRT-PCR
WRKY2P-1363	AACTGCAGACTATAGGGCACGCGTGGTC	Forward primer for promoter deletion
WRKY2P-1018	AACTGCAGTTCTTACGAAAGTGGTGCTGCTA	Forward primer for promoter deletion
WRKY2P-668	AACTGCAGACGCATGTAGCCCTAAACCAAG	Forward primer for promoter deletion
WRKY2P-521	AACTGCAGTTTGCGGTGCTTGAGCTAATT	Forward primer for promoter deletion
WRKY2P-288	AACTGCAGTGTGGTGGATGCTACACCTGAG	Forward primer for promoter deletion
WRKY2P-137	AACTGCAGCATAATGCCCTGTTTCCTCCAA	Forward primer for promoter deletion
WRKY2P-48	AACTGCAGGCAGTGAGTATCCTCGGAGTTAT	Forward primer for promoter deletion
WRKY2-5UTR-F	AACTGCAGAACTAAGAAGAAGGTTGAACGGT	Forward primer for 5′-UTR
WRKY2P-anti	CGGGATCCGGACAATTCAAATGTGTGCACTT	Reverse primer for promoter deletion
WRKY2-5UTR-R	CGGGATCCTCGACGGTTAGGCAGAGGCTTA	Reverse primer for promoter deletion
FWR2PIN	TGAAGCAGGAGAAAGGGCGGAAGGCG	Forward primer for probe
RWR2PIN	CGCTGGTGCCCTTCCATCATCGTCG	Reverse primer for probe

**Table 2:** The WRKY transcription factors in *G. biloba*.

Sequence ID^*∗*^	RPKM		CDs (bp)	Annotation to the Arabidopsis WRKYs
	Male flower	Female flower		
T1_Unigene_BMK.14363	10.97	13.00	2583	WRKY2
CL11186Contig1	21.20	5.54	1977	WRKY4
T1_Unigene_BMK.20451	15.23	38.68	2019	WRKY3
CL8351Contig1	7.37	10.07	2037	WRKY4
CL1197Contig1	7.25	14.22	2325	WRKY20
CL6861Contig1	50.14	3.48	2121	WRKY31
T1_Unigene_BMK.5610	1.92	0.00	783	WRKY61
CL6024Contig1	3.55	2.24	1338	WRKY57
CL10281Contig1	12.46	3.65	744	WRKY28
T1_Unigene_BMK.13300	23.85	18.55	1068	WRKY21
CL7955Contig1	68.18	44.85	1206	WRKY11
T1_Unigene_BMK.24659	7.59	0.45	1281	WRKY42
CL10826Contig1	2.54	1.29	1374	WRKY14
T1_Unigene_BMK.18545	25.53	3.26	642	WRKY16
T2_Unigene_BMK.20037	15.38	39.75	1440	WRKY4
CL7159Contig1	11.59	6.68	1086	WRKY21
T1_Unigene_BMK.7872	3.06	1.19	900	WRKY31
T2_Unigene_BMK.15740	11.05	13.52	1806	WRKY2
CL2657Contig1	2.31	23.10	804	WRKY71
CL3411Contig1	5.22	1.97	1527	WRKY48
T2_Unigene_BMK.8142	21.95	18.36	1068	WRKY21
CL11312Contig1	32.03	14.08	1101	WRKY7
T2_Unigene_BMK.4532	0.08	1.46	258	WRKY12
**T2_Unigene_BMK.10348**	**10.05**	**2.10**	**808**	**WRKY2**
T2_Unigene_BMK.10347	18.22	4.04	915	WRKY2
CL1517Contig1	22.31	6.87	1080	WRKY21
T2_Unigene_BMK.9572	0.13	2.84	1404	WRKY49
CL4085Contig1	1.76	0.69	378	WRKY23

^*∗*^The candidate gene is indicated in bold.

**Table 3:** Putative *cis*-acting regulatory elements identified in the promoter of *GbWRKY2* using PLACE (http://www.dna.affrc.go.jp/PLACE/) and the Signal Scan Program PlantCARE (http://bioinformatics.psb.ugent.be/webtools/plantcare/html/) database.

Name of* cis*-element	Position	Signal sequence	Function	References
ABRELATERD1	585	ACGTG	ABA-responsive elements	[24]

ABRERATCAL	10	MACGYGB	Ca^2+^-responsive and ABA upregulated genes	[25]

ARR1AT	629, 634, 564, 90, 751, 883	NGATT	ARR1 binding element involved in cytokinin signaling	[26]

ACGTATERD1	262, 585	ACGT	Involved in upregulation by dehydration stress and dark-induced senescence	[27]

BIHD1OS	595, 772, 863, 1307	TGTCA	BELL recognition site involved in disease resistance responses	[28]

CAATBOX1	232, 787, 824	CAAT	Common *cis*-acting element in promoter and enhancer regions	[29]

CRTDREHVCBF2	18	GTCGAC	Low-temperature responsive	[30]

DOFCOREZM	354, 621, 687, 1258, 1301	AAAG	Dof1 and Dof2 binding element involved in carbon metabolism	[31]

DPBFCOREDCDC3	1089	ACACNNG	DPBF-1 binding core sequence involved in ABA signaling	[32]

EBOXBNNAPA	86, 232, 318, 714, 729, 775, 1090, 1246, 1280, 1350	CANNTG	*Cis*-element binding BHLH factor is dispensable for light responsiveness	[33]

GATABOX	124, 222, 290, 640, 681, 1253, 1276	GATA	Common *cis*-acting element in promoter	[34]

GT1CONSENSUS	475, 803, 928	GRWAAW	Consensus GT-1 binding site in many light-regulated genes and influence the level of SA-inducible gene expression	[35]

IBOX	124	GATAAG	Conserved sequence upstream of light-regulated genes	[36]

MYB2CONSENSUSAT	318, 1246, 1280, 591	YAACKG	MYB recognition site involved in dehydration responsiveness	[37]

MYCCONSENSUSAT	86, 232, 318, 714, 729, 775, 1090, 1246, 1280	CANNTG	MYC recognition site involved in dehydration responsiveness and cold responsiveness	[38]

PYRIMIDINE-box	214, 840, 869, 890	CCTTTT	Gibberellin-response cis-element	[39]

ROOTMOTIFTAPOX1	178, 223, 546, 641, 722	ATATT	Root specific expression	[40]

TATA box	94	TTATTT	Common *cis*-acting element in promoter and enhancer regions	[41]

WBOXATNPR1	887	TTGACY	WRKY binding site, involved in many physiological processes	[10]
