# Supplementary material for: Isolation, Expression, and Promoter Analysis of GbWRKY2: A Novel Transcription Factor Gene from Ginkgo biloba
Source: Int J Genomics. 2015 Aug 16;2015:607185. doi: 10.1155/2015/607185 (PMC4553201; doi:10.1155/2015/607185)
Supplement: Supplementary file 1 — Figure S1. Nucleotide sequence of the 5'-upstream sequence of GbWRKY2. Various cis-elements were predicted from PlantCARE and PALCE plantforms. Coding region for GbWRKY2 gene is underlined, and translation start site is indicated by a translated methionine under ATG and shadowed. The transcription start site (TSS) and TATA-box are indicated as +1 and −26, respectively. Description of various cis-elements highlighted in the sequence is tabulated in Table 3. [file 607185.f1.doc]

**Figure S1.** Nucleotide sequence of the 5′-upstream sequence of *GbWRKY2*. Various *cis*-elements were predicted from PlantCARE and PALCE plantforms. Coding region for *GbWRKY2* gene is underlined, and translation start site is indicated by a translated methionine under ATG and shadowed. The transcription start site (TSS) and TATA-box are indicated as +1 and -26, respectively. Description of various *cis*-elements highlighted in the sequence is tabulated in Table 3.
